# Human Papillomavirus 16 E6 and E7 Synergistically Repress Innate Immune Gene Transcription

**DOI:** 10.1128/mSphere.00828-19

**Published:** 2020-01-08

**Authors:** Claire D. James, Christian T. Fontan, Raymonde Otoa, Dipon Das, Apurva T. Prabhakar, Xu Wang, Molly L. Bristol, Iain M. Morgan

**Affiliations:** a Philips Institute for Oral Health Research, School of Dentistry, Virginia Commonwealth University, Richmond, Virginia, USA; b Massey Cancer Center, Virginia Commonwealth University, Richmond, Virginia, USA; University of Michigan-Ann Arbor

**Keywords:** DNA damage response, E6, E7, innate immunity, papillomavirus

## Abstract

Human papillomaviruses (HPV) are causative agents in 5% of all cancers, including the majority of anogenital and oropharyngeal cancers. Downregulation of innate immune genes (IIGs) by HPV to promote the viral life cycle is well documented; E6 and E7 are known repressors of these genes. More recently, we demonstrated that E2 could also repress IIGs. These studies have been carried out in cells overexpressing the viral proteins, and to further investigate the role of individual viral proteins in this repression, we introduced stop codons into E6 and/or E7 in the entire HPV16 genome and generated N/Tert-1 cells stably maintaining the HPV16 genomes. We demonstrate that E6 or E7 individually is not sufficient to repress IIG expression in the context of the entire HPV16 genome; both are required for a synergistic repression. The DNA damage response (DDR) is activated by HPV16 irrespective of E6 and E7 expression, presumably due to viral replication; E1 is a known activator of the DDR. In addition, replication stress was apparent in HPV16-positive cells lacking E6 and E7, manifested by attenuated cellular growth and activation of replication stress genes. These studies led us to the following model. Viral replication *per se* can activate the DDR following infection, and this activation is a known inducer of IIG expression, which may induce cellular senescence. To combat this, E6 and E7 synergistically combine to manipulate the DDR and actively repress innate immune gene expression promoting cellular growth; neither protein by itself is able to do this.

**IMPORTANCE** The role of human papillomavirus 16 (HPV16) in human cancers is well established; however, to date there are no antiviral therapeutics that are available for combatting these cancers. To identify such targets, we must enhance the understanding of the viral life cycle. Innate immune genes (IIGs) are repressed by HPV16, and we have reported that this repression persists through to cancer. Reversal of this repression would boost the immune response to HPV16-positive tumors, an area that is becoming more important given the advances in immunotherapy. This report demonstrates that E6 and E7 synergistically repress IIG expression in the context of the entire HPV16 genome. Removal of either protein activates the expression of IIGs by HPV16. Therefore, gaining a precise understanding of how the viral oncogenes repress IIG expression represents an opportunity to reverse this repression and boost the immune response to HPV16 infections for therapeutic gain.

## Abstract (precis)

The role of human papillomavirus 16 (HPV16) in human cancers is well established; however, to date there are no antiviral therapeutics that are available for combatting these cancers. To identify such targets, we must enhance the understanding of the viral life cycle. Innate immune genes (IIGs) are repressed by HPV16, and we have reported that this repression persists through to cancer. Reversal of this repression would boost the immune response to HPV16-positive tumors, an area that is becoming more important given the advances in immunotherapy. This report demonstrates that E6 and E7 synergistically repress IIG expression in the context of the entire HPV16 genome. Removal of either protein activates the expression of IIGs by HPV16. Therefore, gaining a precise understanding of how the viral oncogenes repress IIG expression represents an opportunity to reverse this repression and boost the immune response to HPV16 infections for therapeutic gain.

## INTRODUCTION

Human papillomavirus (HPV) is the most common sexually transmitted infection in the United States, with an estimated 80% of sexually active adults acquiring an HPV infection in their lifetime (1). Of the high-risk HPVs known to be causative in the development of cancer, HPV16 is the most prevalent genotype (2). HPV16 is causative in around 50% of cervical cancers and nearly 90% of HPV-related head and neck squamous cell carcinomas (HPV+HNSCCs). Further understanding of HPV16 and its life cycle is needed in order to develop novel antiviral therapies targeting HPV16.

Much has been discovered relating to the viral oncoproteins E6 and E7 driving cellular growth and promoting infection (3–9). Most well known is the targeting of cellular p53 and pRb by E6 and E7, respectively. E6 facilitates the degradation of p53 through its association with E6AP, a component of the ubiquitin degradation pathway, whereas E7 binds to and disrupts the functions of pRb. The viral life cycle is dependent upon the differentiation program of epithelia; together, E6 and E7 uncouple the process of differentiation and exit from the cell cycle to allow the virus to replicate and generate progeny virus.

In addition to targeting cellular proteins, both E6 and E7 can regulate transcription from the host genome of infected cells (6, 7, 10–12). Among the genes targeted for repression by E6 and E7 are the innate immune genes (IIGs) (11–16). Both viral oncoproteins have been shown to target the expression of these genes, and such repression would promote viral infection by an overall suppression of the host immune response. In these studies, E6 and E7 have been overexpressed from heterologous promoters. HPV16-infected cells also have an active DNA damage response (DDR) turned on at all times; E6, E7, and E1 have all been shown to activate this pathway (17–27). This provides another challenge the virus must overcome, as activation of the DDR stimulates the innate immune response (28). The DDR-mediated activation of the interferon (IFN) response can occur in the absence of detectable foreign nucleic acids, such as after treatment of cells with etoposide. (29). Recently, we demonstrated that repression of IIGs by HPV16 persists in HPV16-positive tumors and that this repression may contribute to the immune evasion by the HPV16 tumor (30, 31). We also recently demonstrated that in addition to E6 and E7, E2 can also repress expression of IIGs (30). Given that all of the IIG studies with E6, E7, and E2 have been done with overexpression of the viral proteins from heterologous promoters, we sought to investigate the contribution of each viral protein to IIG repression in the context of the entire HPV16 genome.

In order to investigate the contribution of the individual viral proteins to IIG repression, we generated mutant HPV16 genomes that had stop codons in E6, E7, or both (see Materials and Methods). These were transfected into the hTERT immortalized foreskin keratinocyte cell line N/Tert-1 to establish stable cell lines containing wild-type HPV16 and the assorted E6/E7 mutants. We have demonstrated that HPV16 represses IIGs in these cells (30, 31). These experiments required the use of already immortalized keratinocytes, as HPV16 cannot immortalize primary keratinocytes in the absence of E6 and E7 expression. The results demonstrate that in the absence of either E6 or E7, there is an activation of IIG expression, not a repression. Removal of expression of both oncoproteins resulted in an additive activation of IIG expression. Strikingly, there is a strong synergism between E6 and E7 to repress IIGs, as both proteins together can repress IIG expression in the context of cells containing the entire HPV16 genome. As HPV16 is known to activate the DDR and an active DDR can activate expression of IIGs (28), we investigated the DDR in the wild-type and mutant HPV16 genomes. We observed that the DDR is active in all HPV16-containing cells, demonstrating that neither E6 nor E7 is required for activation of the DDR by HPV16. This activation likely results from the expression of E1, which is a known activator of the DDR (23, 25–27). We cannot generate stable cell lines overexpressing E1 by itself, as it is toxic to cells. In addition, the cells lacking expression of both E6 and E7 had severely attenuated cellular growth. All cells had activation of genes involved in managing replication stress. The results demonstrate that E6 and E7 synergize to repress expression of IIGs. They also demonstrate that activation of the DDR and induction of replication stress can occur in HPV16 cells independently of E6 and E7 expression, probably due to the replication factor E1.

## RESULTS

### Establishment and characterization of mutant HPV16 genome N/Tert-1 cells.

**FIG 1:**
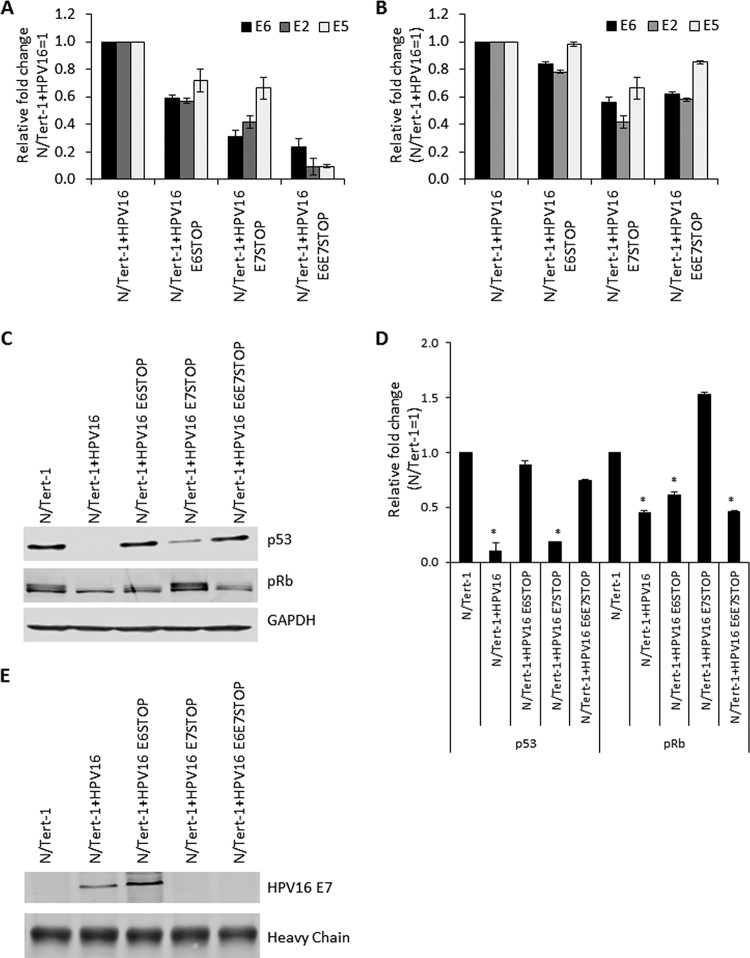
(A) qPCR analysis of DNA extracted from N/Tert-1+HPV16 cell lines. RNase-treated DNA was subject to SYBR green qPCR analysis, and the ΔΔ*C_T_* was calculated using the GAPDH housekeeping gene and normalized to that for N/Tert-1+HPV16. Error bars represent the standard error of the mean of three individual experiments. (B) qRT-PCR analysis of E2, E5, and E6 mRNA expression in N/Tert-1+HPV16 and mutant HPV16 genome-containing cell lines. DNase-treated RNA was subjected to SYBR green qRT-PCR analysis, and the ΔΔ*C_T_* was calculated using the GAPDH housekeeping gene and normalized to that for N/Tert-1+HPV16. Error bars represent the standard error of the mean of three individual experiments. (C) Western blot analysis for p53 and pRb in N/Tert-1, N/Tert-1+HPV16, and mutant HPV16 genome-containing cell lines. GAPDH is shown as a loading control. p53 is downregulated in the presence of wild-type HPV16 and N/Tert-1+HPV16 E7STOP but not in N/Tert-1+HPV16 E6STOP. pRb is downregulated in the presence of wild-type HPV16 and N/Tert-1+HPV16 E6STOP but not in N/Tert-1+HPV16 E7STOP. Both pRb and p53 are unaffected in N/Tert-1+HPV16 E6E7STOP, compared to wild-type HPV16 genome-containing cells. (D) Western blots were visualized, and the results were quantified using a Li-Cor imaging system and calculated in comparison to that for parental N/Tert-1. Data represent the average of three independent experiments, and error bars indicate the standard error of the mean. *, *P* < 0.05, compared to results for parental N/Tert-1 cells. (E) E7 protein expression was confirmed in the N/Tert-1+HPV16 cell lines: E7 was enriched by immunoprecipitation before detection by Western blot analysis.

We introduced stop codons for E6 (residue C110 mutated to a T) or E7 (residue T584 mutated to an A) into the HPV16 genome and both together. We prepared stable pooled N/Tert-1 cell lines containing the wild-type and mutant HPV16 genomes. These were named N/Tert-1+HPV16, N/Tert-1+HPV16 E6 STOP, N/Tert-1+HPV16 E7 STOP, and N/Tert-1+HPV16 E6E7 STOP. The presence of HPV DNA in the cells was confirmed by PCR using E6, E2, and E5 primers (Fig. 1A). While there was less DNA present in the mutants, levels were within 2-fold of each other between the different cell lines. The detection of E6, E2, and E5 demonstrates an intact early region in these cells. We next investigated RNA expression from the viral genome in these cell lines using reverse transcription-quantitative PCR (qRT-PCR) (Fig. 1B). There was less RNA present in the N/Tert-1+HPV16 E6E7 STOP cells, indicating that the E6 and E7 viral oncoproteins may actually promote transcription from the HPV16 genomes. However, in all cell lines, there was significant expression of the viral genes, and again, this was across the entire early region, as E6, E2, and E5 expression is detected.

As markers for E6 and E7 activity, the expression levels of p53 and pRb were investigated (Fig. 1C). Both p53 and pRb are decreased in N/Tert-1+HPV16 cells, and p53 levels are rescued to the same level as the parental N/Tert-1 cells in the E6 STOP and E6E7 STOP genomes. pRb is elevated when E7 is absent (E7 STOP), as would be expected. Surprisingly, pRb was downregulated in N/Tert-1+HPV16 E6/E7 STOP cells. We confirmed that the E7 was mutated in these cells by DNA sequencing to eliminate the possibility of a plasmid mix-up during transfection (not shown). These cells are stressed and have very slow growth (see below); therefore, the presence of the replicating genome in the absence of E6 and E7 targets pRb for downregulation by an as-yet-unknown mechanism. These blot analyses were repeated, and the results were quantitated (Fig. 1D). To confirm that E7 is appropriately expressed in the mutant genomes, we detected the E7 protein, by enriching the protein by immunoprecipitation before immunoblotting (Fig. 1E).

### Absence of E6 and E7 expression increases innate immune gene expression in HPV16-containing cells.

Our previous work in N/Tert-1 cells demonstrates that there is downregulation of innate immune gene expression at various stages of the interferon signaling pathway by HPV16 (30, 31). Following treatment of cells with interferon, there is an activation of ISGF3 (interferon-stimulated gene factor 3), which is a complex composed of STAT1, STAT2, and IRF9 (32, 33). This transcription factor complex then enters the nucleus and binds to the control elements of interferon-stimulated genes (ISGs) to activate their transcription. These ISGs are also downregulated in HPV16-positive N/Tert-1 cells (30, 31). These genes are repressed by E6, E7, and E2, and it was unclear what viral protein is responsible for this repression in the context of the entire HPV16 genome. Therefore, we investigated the expression of ISGs and STAT1-IRF9-STAT2 in the mutant genome HPV16-positive N/Tert-1 cells.

**FIG 2:**
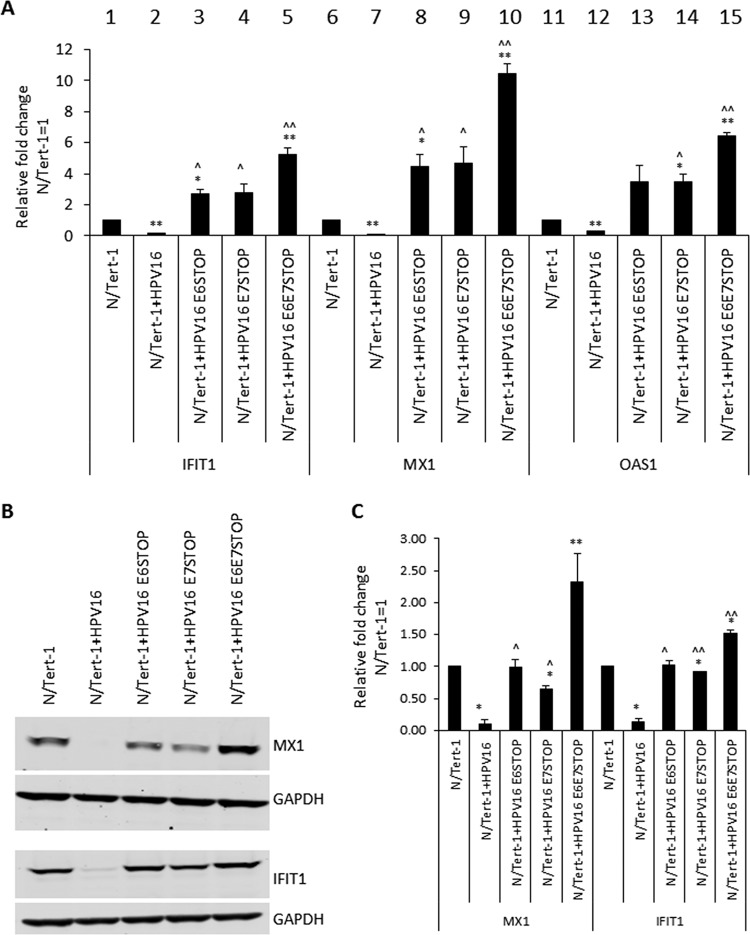
(A) Quantification, by qRT-PCR, of expression of interferon-stimulated genes, namely the IFIT1, MX1, and OAS1 genes, in N/Tert-1, N/Tert-1+HPV16, and mutant genome-containing N/Tert-1 cell lines. The ΔΔ*C_T_* was calculated using the GAPDH housekeeping gene and normalized to that of parental N/Tert-1. Data represent the average of three independent experiments, and error bars indicate the standard error of the mean. *, *P* < 0.05, and **, *P* < 0.001, compared to results for N/Tert-1; ^, *P* < 0.05, compared to results for N/Tert-1+HPV16; ^̂, *P* < 0.001, compared to results for N/Tert-1+HPV16. (B) IFIT1 and MX1 protein expression was observed by Western blot analysis in N/Tert-1 cell lines. Representative images of three individual experiments are shown, and GAPDH was included as a loading control. (C) Western blots were visualized and the results were quantified using a Li-Cor imaging system and calculated in comparison to that for parental N/Tert-1. Data represent the average of three independent experiments, and error bars indicate standard errors of the mean. *, *P* < 0.05, and **, *P* < 0.001, compared to results for N/Tert-1; ^, *P* < 0.05, compared to results for N/Tert-1+HPV16; and ^̂, *P* < 0.001, compared to results for N/Tert-1+HPV16.

Figure 2A demonstrates that the IFIT1, MX1, and OAS1 ISGs are repressed by HPV16 in N/Tert-1 cells, as we have reported (lanes 2, 7, and 12). Strikingly, ablation of E6 expression (lanes 3, 8, and 13) or E7 expression (lanes 4, 9, and 14) elevates ISG expression higher than that in control N/Tert-1 cells (lanes 1, 6, and 11). In addition, ablation of both E6 and E7 expression (lanes 5, 10, and 15) results in an additive increase in ISG expression. The MX1 and IFIT1 proteins were elevated to the level observed in the parental N/Tert-1 cells in the absence of E6 or E7 expression (Fig. 2B). The Western blot analysis shown in Fig. 2B was repeated, and the results were quantitated (Fig. 2C). However, the levels of MX1 and IFIT1 were not correlated with the protein levels, as there was not an increase in the innate immune proteins in the absence of E6 or E7 in comparison with parental N/Tert-1 cells. Preliminary data suggest that these viral oncoproteins can also employ posttranscriptional mechanisms to regulate the expression of the innate immune proteins, and this is under active study (not shown). These results demonstrate that both the RNA and protein levels of ISGs are elevated in HPV16-containing N/Tert-1 cells that have abrogated E6 and E7 expression, in comparison with cells containing the wild-type HPV16 genome.

**FIG 3:**
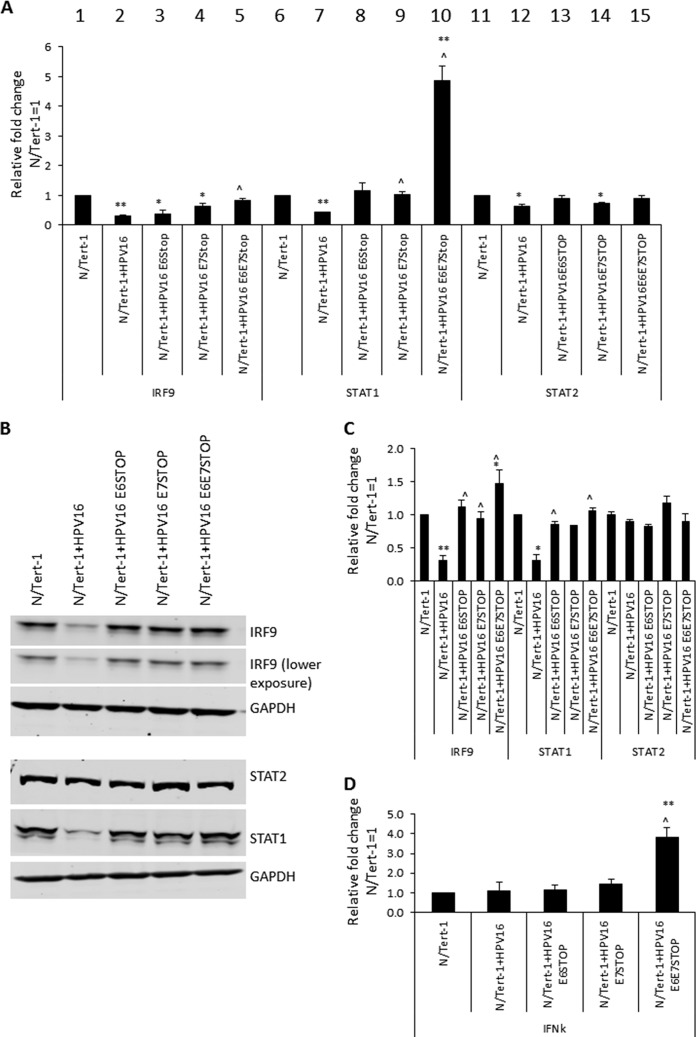
(A) Quantification, by qRT-PCR, of expression of interferon-stimulated genes, namely the IRF9, STAT1, and STAT2 genes, in N/Tert-1, N/Tert-1+HPV16, and mutant genome-containing N/Tert-1 cell lines. The ΔΔ*C_T_* was calculated using the GAPDH housekeeping gene and normalized to that of parental N/Tert-1. Data represent the average of three independent experiments, and error bars indicate the standard error of the mean. *, *P* < 0.05, and **, *P* < 0.001, compared to results for N/Tert-1; ^, *P* < 0.05, compared to results for N/Tert-1+HPV16. (B) IRF9, STAT1, and STAT2 protein expression was observed by Western blot analysis in N/Tert-1 cell lines. Representative images of three individual experiments are shown, and GAPDH was included as a loading control. (C) Western blots were visualized, and results were quantified using a Li-Cor imaging system and calculated in comparison to that for parental N/Tert-1. Data represent the average of three independent experiments, and error bars indicate the standard error of the mean. *, *P* < 0.05, and **, *P* < 0.001, compared to results for N/Tert-1; ^, *P* > 0.05, compared to results for N/Tert-1+HPV16. (D) IFN-κ expression quantified by qRT-PCR. The ΔΔ*C_T_* was calculated using the GAPDH housekeeping gene and normalized to that of parental N/Tert-1. Data represent the average of three independent experiments, and error bars indicate the standard error of the mean. **, *P* < 0.001, compared to results for N/Tert-1.

We looked at the expression of the ISGF3 components in the mutant cells (Fig. 3). As previously reported, STAT1 and IRF9 are repressed by HPV16 in N/Tert-1 cells, while STAT2 is not (Fig. 3A, compare lanes 2, 7, and 12 with lanes 1, 6, and 11) (30, 31). Removal of E6 or E7 abrogates HPV16 repression of STAT1 or IRF9 in N/Tert-1 cells. STAT1 RNA levels were elevated in the absence of both E6 and E7 expression (compare lane 10 with lane 7). Protein expression levels of STAT1 and IRF9 are similarly regulated (Fig. 3B). Any repression of these proteins is lost following abrogation of E6 or E7 expression. The experiment in Fig. 3B was repeated, and the Western blot results were quantitated (Fig. 3C). Unlike the large increase in STAT1 RNA (Fig. 3A), there was no statistically significant difference in STAT1 protein levels in the absence of both E6 and E7 or with abrogation of either protein expression by itself.

To determine whether the activation of STAT1 expression in the absence of E6 and E7 was due to an increase in interferon production, we monitored the RNA levels for interferon genes. There was no detectable expression of alpha or beta interferon (IFN-α or IFN-β) in any of the N/Tert-1 cells. However, there was an increase in IFN-κ RNA in the absence of both E6 and E7 expression, in comparison with parental cells (Fig. 3D, IFN-κ). We previously observed a slight repression of IFN-κ in HPV16-positive N/Tert-1 cells that we did not observe here, perhaps due to the earlier use of clonal lines rather than the pools used here. It is possible that the elevated IFN-κ is responsible for the activation of STAT1 expression.

### The absence of functional E6 and E7 in HPV16-positive N/Tert-1 cells attenuates cellular growth.

**FIG 4:**
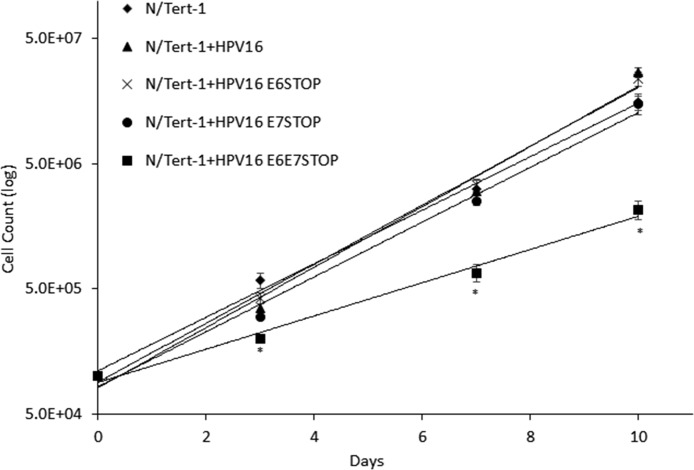
To measure cell growth, cells were seeded in triplicate onto 10-cm dishes at a density of 3 × 10^5^ cells per dish and grown to 80% confluence (typically 3 days). Cells were then harvested by trypsinization and stained with trypan blue, and viable cells were counted. A total of 3 × 10^5^ cells per dish were replated, and this was carried out in triplicate. Data represent the average of three independent experiments, and error bars indicate the standard error of the mean. N/Tert-1+HPV16 cells grew significantly faster than N/Tert-1 cells, and the loss of E6 did not affect this. Removal of E7 resulted in the N/Tert-1 cells growing similarly to the parental N/Tert-1 cells. Removal of E6 and E7 resulted in significantly slower growth of N/Tert-1 cells than of all other lines. In all analyses, a *P* value of <0.05 indicated significance.

In doing the above-described experiments, we noticed that the N/Tert-1+HPV16 E6E7 STOP cells grew slower than the other N/Tert-1 cell lines. To quantitate this, we carried out a growth curve analysis and demonstrated that this was indeed the case (Fig. 4). The presence of the HPV16 genome and the absence of E6/E7 expression resulted in dramatically attenuated growth in comparison with that of parental N/Tert-1 cells. The N/Tert-1+HPV16 cells grew faster than parental N/Tert-1 cells as expected, and removal of E6 expression did not change this increased expression. The absence of E7 expression reduced the growth rate of the cells to that of the parental N/Tert-1 cells. However, the absence of both E6 and E7 synergized to dramatically reduce the growth of the N/Tert-1 cells.

### The DNA damage response is activated in N/Tert-1 cells irrespective of E6/E7 expression.

**FIG 5:**
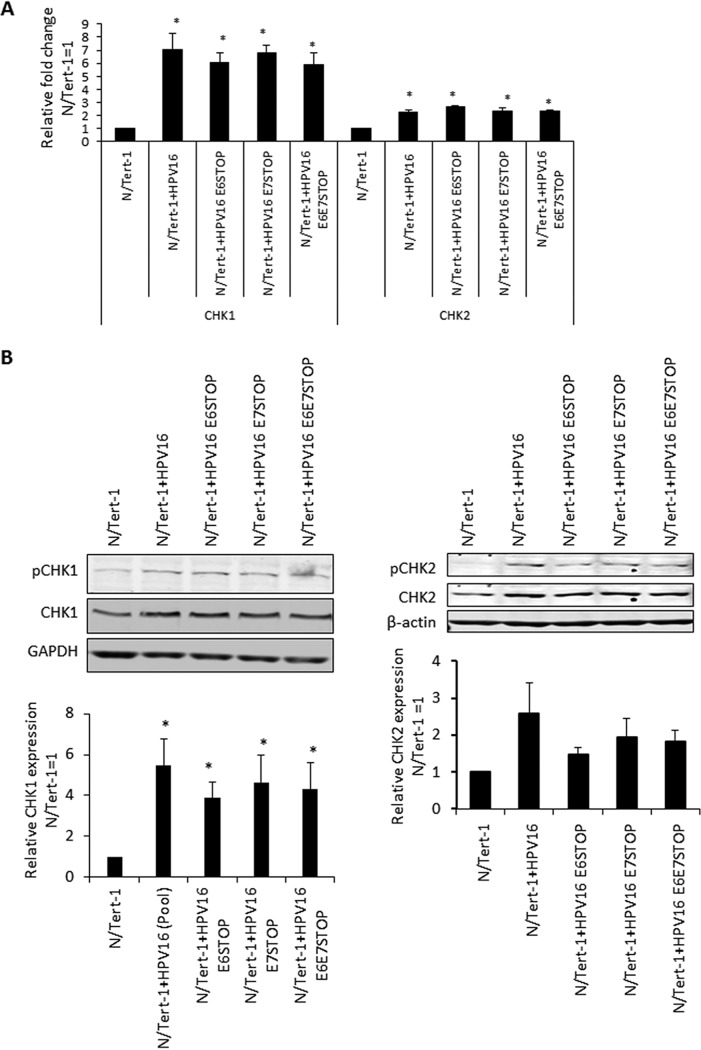

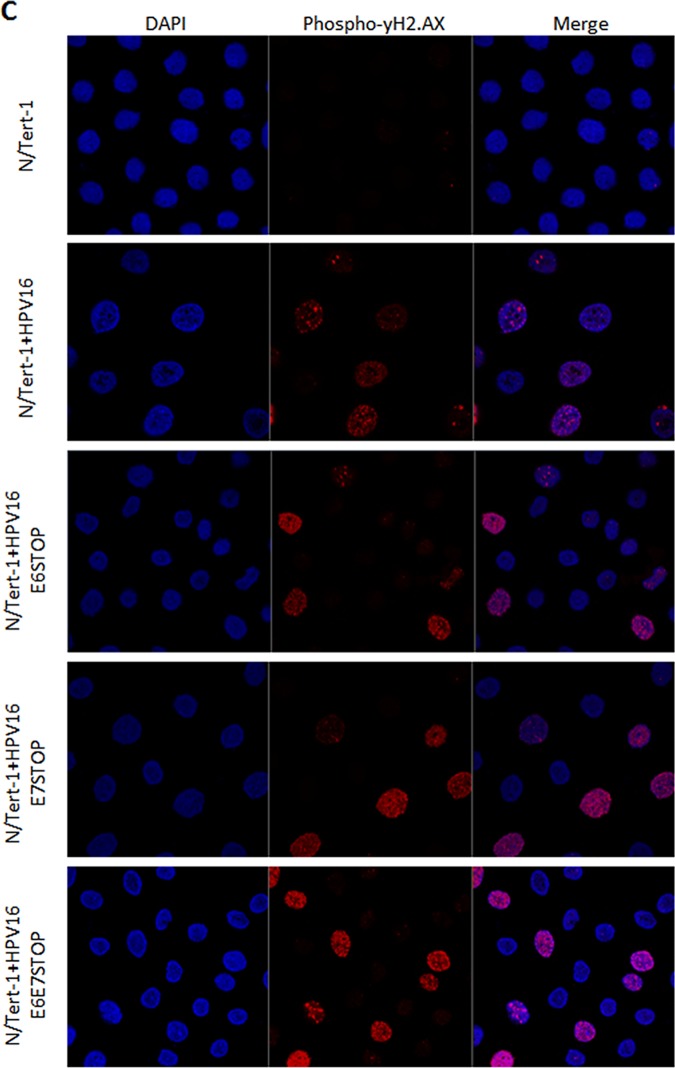
(A) qRT-PCR analysis of CHK1 and CHK2 mRNA expression in N/Tert-1, N/Tert-1+HPV16, and mutant HPV16 genome-containing cell lines. DNase-treated RNA was subject to SYBR green qRT-PCR analysis, and the ΔΔ*C_T_* was calculated using the GAPDH housekeeping gene and normalized to that for N/Tert-1. Error bars represent the standard error of the mean of three individual experiments. *, *P* < 0.05, compared to results for N/Tert-1. (B) CHK1 and CHK2 protein expression and activation (observed as phosphorylated proteins) detected by Western blot analysis of N/Tert-1 cell lines. Representative images of three individual experiments are shown, and GAPDH was included as a loading control. Western blots were visualized, and the results were quantified using a Li-Cor imaging system and calculated relative to that for parental N/Tert-1. Data represent the average of three independent experiments, and error bars indicate the standard error of the mean. *, *P* < 0.05, **, *P* < 0.001, compared to results for N/Tert-1; ^, *P* > 0.05, compared to results for N/Tert-1+HPV16. (C) Levels of DNA damage indicator phospho-γ-H2AX assessed by immunofluorescent staining of monolayer-grown cells. Prior to staining, N/Tert-1 cell lines were grown to 70% confluence. Cellular DNA was stained by inclusion of 4′,6-diamidino-2-phenylindole (DAPI). Images are representative of three individual experiments.

It is well established that HPV16 activates the DNA damage response (DDR) in keratinocytes and that individual overexpression of E6 or E7 can also activate the DDR (17, 18, 20–22, 24, 34, 35). In addition, the E1 protein has also been shown to activate the DDR (23, 25–27). Given that activation of the DDR pathway in non-HPV16-containing cells ordinarily results in growth attenuation, we investigated whether the DDR was activated in HPV16 cells in the absence of expression of either E6 or E7 or both. The levels of CHK1 and CHK2 RNA in N/Tert-1 cells are elevated by the HPV16 genome irrespective of E6/E7 status (Fig. 5A). This is reflected at the protein level (Fig. 5B). In addition, both CHK1 and CHK2 are activated in HPV16-positive N/Tert-1 cells irrespective of E6 and E7 status, as demonstrated by an increase in activating phosphorylation of these proteins. Using immunofluorescence staining, we also observed that levels of γ-H2AX are increased in N/Tert-1 cells containing HPV16 irrespective of E6 and E7 levels (Fig. 5C).

**FIG 6:**
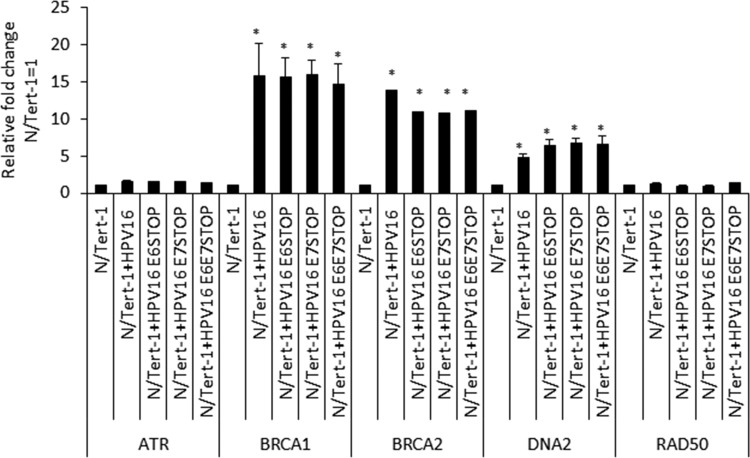
qRT-PCR analysis of DNA damage response genes in N/Tert-1, N/Tert-1+HPV16, and mutant HPV16 genome-containing cell lines. DNase-treated RNA was subject to SYBR green qRT-PCR analysis, and the ΔΔ*C_T_* was calculated using the GAPDH housekeeping gene and normalized to that for N/Tert-1. Error bars represent the standard error of the mean of three individual experiments. *, *P* < 0.05, compared to results for parental N/Tert-1 cells.

These results suggested that the N/Tert-1 cells containing HPV16 were under replication stress irrespective of E6/E7 status of the viral genome. We next investigated the RNA expression pattern of stress response genes in the N/Tert-1 cells containing the HPV16 genomes (Fig. 6). BRCA1, BRCA2, and DNA2 levels were all elevated in the HPV16-positive N/Tert-1 cells compared with the parental control, irrespective of E6 and E7 status. For ATR and Rad50, there were less dramatic increases in expression by any of the HPV16 genomes. The results investigating the DDR pathway in the N/Tert-1 cells containing the HPV16 genome demonstrate that replication stress and activation of the DDR are turned on by HPV16 independently of E6 and E7 expression.

## DISCUSSION

Several laboratories have demonstrated repression of the innate immune response by high-risk HPV in keratinocytes (13–16). It has also been established that when E6 and E7 are overexpressed individually in keratinocytes, they can repress expression of innate immune genes (IIGs) (12, 36–38). Recently, we demonstrated that HPV16 E2 can also repress IIG expression when overexpressed in keratinocytes (30). Given that all three viral proteins can repress innate immune gene expression when overexpressed, we sought to extend these studies to the HPV16 genome. To do this, we generated keratinocytes containing HPV16 with stop codons in either E6 or E7, or both together, to abrogate oncoprotein expression, and leaving the expression of E2 intact. Given that E6 and E7 are required for the generation of immortalized HPV16-containing primary cells, we used foreskin keratinocytes immortalized by hTERT, N/Tert-1 cells, for these experiments (39). Previously, we have used these cells to demonstrate that E2 can regulate expression of host genes in keratinocytes and that this regulation was relevant to the viral life cycle (30). The mutant and wild-type genome-containing cell lines generated demonstrated the presence of HPV16 DNA with corresponding RNA expression from the viral genome. The N/Tert-1 cells with genomes containing stop codons in E6 or E7 demonstrated the predicted expression of the tumor suppressor proteins p53 and pRb; i.e., p53 is not degraded when E6 is nonfunctional, and pRb is expressed when E7 is nonfunctional. However, in the cells containing the genomes with stop codons in both E6 and E7, the levels of Rb are downregulated by an as-yet-unclear mechanism perhaps related to the replication stress these cells are under (Fig. 1).

We next investigated the expression of interferon-stimulated genes (ISGs) in these cell lines and made several striking observations. First, HPV16 represses the expression of ISGs in N/Tert-1 cells, as we have previously reported (30, 31). Second, in the absence of either E6 or E7, there is an increase in the expression of the ISGs investigated to levels above what is observed in the vector control N/Tert-1 cells. Third, in the absence of both E6 and E7, there is an additive increase in the expression of the ISGs, increasing all of their expression levels to statistically significant higher levels than that in the parental N/Tert-1 cells. Fourth, this increase in RNA expression is reflected in protein expression: elevated levels of IFIT1 and MX1 in the absence of E6 and E7 were observed in comparison with that in parental cells. Fifth, looking at the upstream ISGF3 complex genes (the complex responsible for activating the ISGs), there is an enhanced increase in STAT1 levels in comparison to that in N/Tert-1 cells, where E6 and E7 are not expressed, although this is not reflected at the protein level. Sixth, there is an increased expression of IFN-κ in the absence of both E6 and E7 in comparison with that in all other N/Tert-1 cell lines.

These results demonstrate that in the absence of E6 and E7, E2 cannot repress IIG expression alone. However, neither E6 nor E7 can repress these genes by themselves; therefore, there remains the possibility that E2 synergizes with E6 and E7 to repress IIGs in the context of the entire HPV16 genome. It is also possible that E2, E6, and E7 have roles to play in IIG repression during the different stages of the viral life cycle, and this possibility is under investigation.

It is clear that both E6 and E7 are required to suppress innate immune gene expression in the context of the entire HPV16 genome. While both proteins can repress genes when overexpressed individually, abrogation of expression of either one results in a loss of innate immune gene repression (Fig. 2 and 3). This demonstrates a previously unidentified synergism between the two viral proteins in the context of the entire HPV16 genome. Both proteins have been shown to target the interferon signaling pathway using different mechanisms. E7 can bind to and inhibit the transactivating function of IRF1 (40), while E6 binds to IRF3 and inhibits its transcriptional activity (41) and can also interfere with JAK-STAT activation by binding Tyk2 (36). Our studies demonstrate that abrogation of both E6 and E7 expression results in an additive increase in the expression of ISGs. Therefore, the cross talk between E6 and E7 to suppress the innate immune response is synergistic, and the additive nature of the increase in ISG expression in the absence of these two proteins suggests independent pathways that are targeted by these viral proteins. The results presented here suggest that disruption of the mechanism that either of the viral proteins uses to suppress the innate immune response is a potential therapeutic target that would boost the host immune response against HPV infections.

The next question we addressed was what could be responsible for the elevated innate immune gene expression in the absence of E6/E7? The innate immune response is activated by detection of cytoplasmic DNA via the cGAS-STING pathway, and cytoplasmic DNA can be detected in cells treated with DNA-damaging agents (42). However, HPV16 E7 acts to combat activation of the innate immune response via the cGAS-STING pathway (43). A source for activation of the DDR in HPV16 cells lacking E6 and E7 is the E1 viral helicase that can activate the DDR by itself (23, 25, 26, 44). Although E6 and E7 can activate the DDR when overexpressed in cells, it is clear that in their absence, HPV16 activates DDR pathways, and we propose that this is mediated by the viral replication factor E1 (Fig. 5 and 6). This activation of the DDR by HPV16 replication in the absence of E6 and E7 results in activation of the expression of innate immune response genes, perhaps via activation of the cGAS-STING pathway, although we could not detect several markers for activation of this pathway in the N/Tert-1+HPV16 E6/E7 STOP cells (not shown). In support of the idea that there is increased aberrant DNA sensing in the absence of E6 and E7, levels of IFN-κ are elevated in these cells (Fig. 3). In addition to activation of the DDR, the N/Tert-1+HPV16 E6/E7 STOP cells are under replication stress, as demonstrated by an attenuation of cellular growth (Fig. 4) and activation of expression of genes involved in replication stress (Fig. 6). These same genes can be activated by E7 when it is overexpressed by itself (18). Therefore, there are multiple mechanisms that the virus can use to activate the DDR.

All together, the results presented here prompt us to propose the following model. Following infection, the viral genome copy number increases from 1 to around 50 copies per cell. This increase in copy number would by itself activate the DDR due to torsional stress on the viral DNA. This activation promotes recruitment of homologous recombination factors to the viral genome, a process required for HPV16 replication (45). The activation of the DDR by external DNA-damaging agents results in cell cycle arrest to allow detection of damage and repair of the DNA prior to restarting the cell cycle. HPV16 must circumvent this, as it would prevent amplification of the viral genome. Therefore, E6 and E7 manipulate constituents of the DDR to allow cell cycle progression in the presence of an active DDR (this has been previously demonstrated [17, 18, 34, 35]), thus preventing cell cycle arrest. Simultaneously, the actions of E6 and E7 repress innate immune response genes, and this not only promotes the viral life cycle via immune evasion but also abrogates the growth inhibitory activity of innate immune signaling. We show, for the first time, that this targeting of the innate immune response by E6 and E7 is totally synergistic in the context of the entire HPV16 genome; neither protein is able to do this by itself. Therapeutically, targeting only one of the pathways used by E6 or E7 would potentially alleviate the repression of the innate immune response and boost the host immune response to HPV16 infection.

## MATERIALS AND METHODS

### Generation of HPV16 mutant genomes.

Mutations to introduce T584A (E6 STOP), C110T (E7 STOP), or both C110T and T584A (E6E7 STOP) into HPV16 in the peGFP-N1HPV16 plasmid were carried out by Genscript. Briefly, the target area was released from the rest of the plasmid by EcoNI and NotI restriction digestion, base changes were introduced and amplified via PCR, and then the target area was religated into the plasmid backbone. Successful mutagenesis was confirmed by sequencing. These mutations resulted in (i) an early stop codon in the E6 gene, (ii) an early stop codon in the E7 gene, or (iii) an early stop codon in both the E6 and E7 genes (Fig. 5).

### Cell culture.

Cell lines containing the HPV16 genomes were generated using N/Tert-1 cells as previously described (30, 31). These cells were cultured alongside parental N/Tert-1 cells for all comparisons. N/Tert-1 and N/Tert-1+HPV16 cells were grown in K-SFM (Invitrogen) with a 1% (vol/vol) penicillin-streptomycin mixture (Thermo Fisher Scientific) containing 4 μg/ml hygromycin B (Millipore Sigma) at 37°C in a 5% CO_2_–95% air atmosphere and passaged every 3 to 4 days. Cells containing wild-type or mutant HPV16 were grown in the same medium but also containing 150 μg/ml G418 (Thermo Fisher Scientific). All cells were routinely checked for mycoplasma contamination.

To measure cell growth, cells were seeded in triplicate onto 10-cm dishes at a density of 3 × 10^5^ cells per dish and grown to 80% confluence (typically 3 days). Cells were then harvested by trypsinization and stained with trypan blue, and viable cells were counted. A total of 3 × 10^5^ cells per dish were replated, and this was repeated three times in total.

For downstream protein and nucleic acid analysis, 1 × 10^6^ cells were plated onto 100-mm plates, trypsinized and pelleted after 24 h, and washed twice with phosphate-buffered saline (PBS).

### RNA and DNA analysis.

RNA was isolated using the SV total RNA isolation system (Promega) by following the manufacturer’s instructions, including the DNase treatment step. Two micrograms of RNA was reverse transcribed into cDNA using a high-capacity reverse transcription kit (Applied Biosystems). cDNA and relevant primers were added to PowerUp SYBR green master mix (Applied Biosystems), and real-time PCR was performed using a 7500 fast real-time PCR system. Primer sequences (all 5′ to 3′) were as follows: HPV16 E2 F, ATGGAGACTCTTTGCCAACG; HPV16 E2 R, TCATATAGACATAAATCCAG; HPV16 E6 F, TTGAACCGAAACCGGTTAGT; HPV16 E6 R, GCATAAATCCCGAAAAGCAA; HPV16 E5 F, CACAACATTACTGGCGTGCT; HPV16 E5 R, ACCTAAACGCAGAGGCTGCT; GAPDH F, GGAGCGAGATCCCTCCAAAAT; GAPDH R, GGCTGTTGTCATACTTCTCATGG; IRF9 F, GCCCTACAAGGTGTATCAGTTG; IRF9 R, TGCTGTCGCTTTGATGGTACT; STAT1 F, CAGCTTGACTCAAAATTCCTGGA; STAT1 R, TGAAGATTACGCTTGCTTTTCCT; STAT2 F, CCAGCTTTACTCGCACAGC; STAT2 R, AGCCTTGGAATCATCACTCCC; IFN-k F, GTGGCTTGAGATCCTTATGGGT; IFN-k R, CAGATTTTGCCAGGTGACTCTT; IFIT1 F, AGAAGCAGGCAATCACAGAAAA; IFIT1 R, CTGAAACCGACCATAGTGGAAAT; MX1 F, GGTGGTCCCCAGTAATGTGG; MX1 R, CGTCAAGATTCCGATGGTCCT; CHK1 F, ATATGAAGCGTGCCGTAGACT; CHK1 R, TGCCTATGTCTGGCTCTATTCTG; CHK2 F, TCTCGGGAGTCGGATGTTGAG; CHK2 R, CCTGAGTGGACACTGTCTCTAA; ATR F, GGCCAAAGGCAGTTGTATTGA; ATR R, GTGAGTACCCCAAAAATAGCAGG; BRCA1 F, TTGTTACAAATCACCCCTCAAGG; BRCA1 R, CCCTGATACTTTTCTGGATGCC; BRCA2 F, ACAAGCAACCCAAGTGTCAAT; BRCA2 R, TGAAGCTACCTCCAAAACTGTG; RAD50 F, TACTGGAGATTTCCCTCCTGG; RAD50 R, AGACTGACCTTTTCACCATGC; OAS1 F, TGTCCAAGGTGGTAAAGGGTG; OAS1 R, CCGGCGATTTAACTGATCCTG.

DNA was extracted from monolayer-grown cells by lysis in HIRT lysis buffer (400 mM NaCl, 10 mM Tris-HCl, 10 mM EDTA). Cell extracts were incubated with 50 g/ml RNase A, and with proteinase K sequentially to remove residual RNA and proteins, followed by phenol-chloroform extraction. DNA was resuspended in Tris-EDTA (TE) buffer and quantitated by spectrophotometry. Following dilution, 10 ng of DNA and relevant primers were added to PowerUp SYBR green master mix (Applied Biosystems) and real-time PCR was performed using a 7500 fast real-time PCR system, using SYBR green reagent. Primer sequences were as follows: HPV16 E2 F, 5′-ATGGAGACTCTTTGCCAACG-3′; HPV16 E2 R, 5′-TCATATAGACATAAATCCAG-3′; HPV16 E6 F, 5′-TTGAACCGAAACCGGTTAGT-3′; HPV16 E6 R, 5′-GCATAAATCCCGAAAAGCAA-3′; HPV16 E5 F, 5′-CACAACATTACTGGCGTGCT-3′; HPV16 E5 R, 5′-ACCTAAACGCAGAGGCTGCT-3′.

### Protein analysis.

A total of 1 × 10^6^ cells were lysed in 50 μl NP-40 lysis buffer (0.5% Nonidet P-40, 50 mM Tris, pH 7.8, 150 mM NaCl) supplemented with protease inhibitor (Roche Molecular Biochemicals) and phosphatase inhibitor cocktail (Sigma). The cell and lysis buffer mixture was incubated on ice for 20 min and centrifuged for 20 min at 184,000 relative centrifugal force (rcf) at 4°C, and the supernatant was collected. Protein levels were determined utilizing the Bio-Rad protein estimation assay. Equal amounts of protein were boiled in 2× Laemmli sample buffer (Bio-Rad). Samples were then loaded into a Novex 4-to-12% gradient Tris-glycine gel (Invitrogen), run at 100 V for approximately 2 h, and then transferred onto nitrocellulose membranes (Bio-Rad) at 30 V overnight using the wet blot method. Membranes were blocked in Odyssey blocking buffer (diluted 1:1 with PBS) at room temperature for 6 h and probed with relevant antibody diluted in Odyssey blocking buffer overnight at 4°C. Membranes were then washed with PBS supplemented with 0.1% Tween (PBS-Tween) before probing with the corresponding Odyssey secondary antibody (goat anti-mouse IRdye800cw or goat anti-rabbit IRdye680cw) diluted 1:10,000 for 1 h at 4°C. Membranes were washed in PBS-Tween before infrared scanning using the Odyssey CLx Li-Cor imaging system. The antibodies to the following proteins were used for Western blot analysis at 1:1,000 dilutions in Odyssey blocking buffer, diluted 1:1 with PBS: IFIT1 (D2X9Z), MX1 (D3W7I), and IRF9 (D8G7H) from Cell Signaling Technology and STAT1 (sc-346), STAT2 (sc-1668), pSTAT1 Tyr 701 (sc-135648), pRb (SC-102), p53 (sc-47698), and GAPDH (sc-47724) from Santa Cruz Biotechnology.

For immunoprecipitation of E7, 0.5 mg of total protein (extracted as described above) was incubated overnight with E7 antibody (8C9; Thermo Fisher Scientific) at 4°C with rotation in a total volume of 500 μl. This was then incubated for 8 h with prewashed protein A-Sepharose 4B fast flow beads (Sigma). The lysate-bead mixture was rotated at 4°C for 6 h, and the beads were washed five more times with lysis buffer to remove nonspecific protein binding. The beads were then boiled in 4× Laemmli sample buffer (Bio-Rad), and the supernatant of this mixture was loaded into a Novex 4-to-12% gradient Tris-glycine gel (Invitrogen) and treated as a Western blot, as described above.

### Immunofluorescence.

Cells were grown on coverslips to 70% confluence, fixed with formaldehyde, and permeabilized with NP-40. Cells were incubated with the primary antibody for 1 h (phospho-yH2.AX; Cell Signaling Technology 20E3; 1/500), diluted in 10% normal goat serum. Coverslips were washed three times in PBS-Tween (0.1% Tween), and immune complexes were visualized using Alexa 488- or Alexa 595-labeled anti-species-specific antibody conjugates (Molecular Probes). Cellular DNA was stained by inclusion of 4′,6-diamidino-2-phenylindole (DAPI; Santa Cruz sc-3598) in the penultimate wash. Microscopy was performed at the VCU Microscopy Facility. Immunofluorescence was observed using an LSM 700 laser scanning microscope and ZEN 2011 software (Carl Zeiss). Images were assembled in Adobe Photoshop CS 6.0.

### Statistics.

The standard error was calculated from at least three independent experiments, and significance was determined using Student's *t* test.
